# A Single Point Mutation Controls the Rate of Interconversion Between the *g* ^*+*^ and *g* ^*−*^ Rotamers of the Histidine 189 χ2 Angle That Activates Bacterial Enzyme I for Catalysis

**DOI:** 10.3389/fmolb.2021.699203

**Published:** 2021-07-08

**Authors:** Jeffrey A. Purslow, Jolene N. Thimmesch, Valeria Sivo, Trang T. Nguyen, Balabhadra Khatiwada, Rochelle R. Dotas, Vincenzo Venditti

**Affiliations:** ^1^ Department of Chemistry, Iowa State University, Ames, IA, United States; ^2^ Department of Environmental, Biological and Pharmaceutical Sciences and Technologies, Università Degli Studi Della Campania, Caserta, Italy; ^3^ Roy J. Carver Department of Biochemistry, Biophysics and Molecular Biology, Iowa State University, Ames, IA, United States

**Keywords:** NMR, conformational dynamics, thermophile, protein design, enzyme regulation

## Abstract

Enzyme I (EI) of the bacterial phosphotransferase system (PTS) is a master regulator of bacterial metabolism and a promising target for development of a new class of broad-spectrum antibiotics. The catalytic activity of EI is mediated by several intradomain, interdomain, and intersubunit conformational equilibria. Therefore, in addition to its relevance as a drug target, EI is also a good model for investigating the dynamics/function relationship in multidomain, oligomeric proteins. Here, we use solution NMR and protein design to investigate how the conformational dynamics occurring within the N-terminal domain (EIN) affect the activity of EI. We show that the rotameric *g* ^*+*^-to-*g* ^*−*^ transition of the active site residue His^189^ χ2 angle is decoupled from the state A-to-state B transition that describes a ∼90° rigid-body rearrangement of the EIN subdomains upon transition of the full-length enzyme to its catalytically competent closed form. In addition, we engineered EIN constructs with modulated conformational dynamics by hybridizing EIN from mesophilic and thermophilic species, and used these chimeras to assess the effect of increased or decreased active site flexibility on the enzymatic activity of EI. Our results indicate that the rate of the autophosphorylation reaction catalyzed by EI is independent from the kinetics of the *g* ^*+*^-to-*g* ^*−*^ rotameric transition that exposes the phosphorylation site on EIN to the incoming phosphoryl group. In addition, our work provides an example of how engineering of hybrid mesophilic/thermophilic chimeras can assist investigations of the dynamics/function relationship in proteins, therefore opening new possibilities in biophysics.

## Introduction

Enzyme I (EI) is the first protein in the bacterial phosphotransferase system (PTS), a signal transduction pathway that controls multiple cellular functions including sugar uptake, catabolic gene expression, interactions between carbon and nitrogen metabolisms, chemotaxis, biofilm formation, and virulence, *via* phosphorylation-dependent protein-protein interactions (Postma et al., 1993; Clore and Venditti, 2013). The phosphorylation state of EI dictates the phosphorylation state of all other downstream components of the PTS (Deutscher et al., 2014) and malfunction of EI has been linked to reduced growth-rate and attenuated virulence in several bacterial species (Edelstein et al., 1999; Jones et al., 2000; Lau et al., 2001; Kok et al., 2003). Given its central role in controlling bacterial metabolism, EI has been proposed as a target for antimicrobial design (Kok et al., 2003; Huang et al., 2013; Nguyen and Venditti, 2020) or for metabolic engineering efforts aimed at developing more efficient systems for microbial production of chemicals from biomass feedstocks (Doucette et al., 2011; Venditti et al., 2013).

**FIGURE 1:**
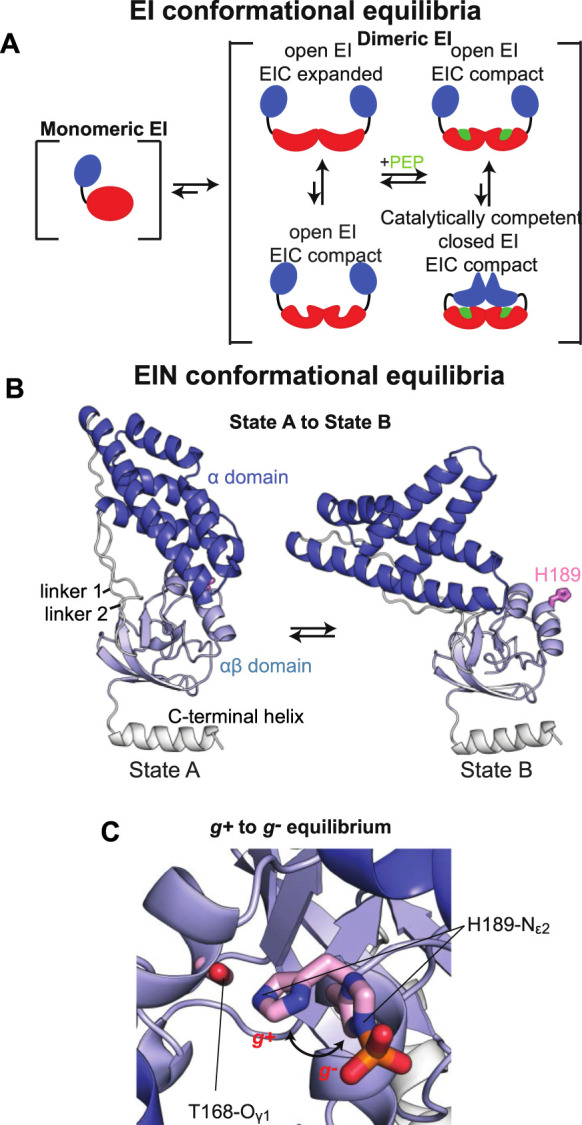
Conformational equilibria of EI during catalysis. **(A)** Schematic summary of the EI conformational equilibria during catalysis. The EIN domain is colored blue, the EIC domain is colored red, the PEP molecule is colored green **(B)** The EIN domain adopts the state A and state B conformation in open (PDB code: 2KX9) and closed (PDB code: 2HWG) EI, respectively. The α and α/β subdomains of EIN are colored blue and light blue, respectively. The active site His^189^ is shown as pink sticks. The linkers connecting the EIN subdomains and the helical linker connecting EIN to EIC are colored white. **(C)** The active site His^189^ adopts the *g* ^*+*^ and *g−* rotameric states in the structures of unphosphorylated and phosphorylated EI, respectively. The α and α/β subdomains of EIN are colored blue and light blue, respectively. The side chains of Thr^168^ and of unphosphorylated and phosphorylated His^189^ are shown as sticks (carbon is pink, nitrogen is blue, oxygen is red, and phosphorus is orange).

In addition to its relevance for pharmaceutical and biotech applications, EI is an ideal model system for investigating the interplay between ligand binding, post-translational modifications, and conformational dynamics that determine the activity of complex multidomain proteins. Indeed, EI is a 128 kDa dimeric enzyme (Chauvin and Brand, 1996) whose activity depends on the synergistic action of at least four conformational equilibria that results in a series of large intradomain, interdomain, and intersubunit structural rearrangements modulated by substrate binding and two subsequent protein phosphorylation steps (from the substrate to EI and from EI to HPr, the second protein of the PTS) (Figure 1A). The N-terminal phosphoryl-transfer domain (EIN, residues 1–249) contains the phosphorylation site (His^189^) and the binding site for the phosphocarrier protein, HPr. The C-terminal domain (EIC, residues 261–575) is responsible for dimerization and contains the binding site for the substrate phosphoenolpyruvate (PEP) and the small molecule regulator α-ketoglutarate (αKG) (Chauvin and Brand, 1996; Venditti et al., 2013). A long helical linker connects the EIN and EIC domains. In the absence of substrate, EI adopts an open conformation in which the EIN domains of the two monomeric subunits are more than 60 Å apart (Schwieters et al., 2010). Binding of PEP induces a transition to the catalytically competent closed form of EI (Venditti et al., 2015a; Venditti et al., 2015b). In the closed structure, the EIN domains of the two monomeric subunits are in direct contact and the active site residue, His^189^, is inserted in the catalytic pocket on EIC (Figure 1A) (Teplyakov et al., 2006).

In recent years, we have published several studies revealing that progressive quenching of the intradomain EIC dynamics is an important source of functional regulation that can be exploited to design allosteric inhibitors of EI. In particular, by using high-pressure NMR we have shown that dimerization of EI promotes substrate binding by providing structural stabilization to the EIC catalytic pocket (Nguyen et al., 2021). Coupling NMR relaxation experiments with Small Angle X-ray Scattering, we showed that binding of PEP results in further quenching of μs-ms dynamics at the EIC catalytic loops that triggers the open-to-close interdomain rearrangement and activates EI for catalysis (Venditti et al., 2015b). Finally, by combining NMR with Molecular Dynamics (MD) simulations, we noticed that residual conformational heterogeneity at the EIC active site in the activated enzyme-substrate complex determines the enzymatic turnover (Dotas et al., 2020) and that perturbing conformational dynamics at the active site loops is an effective strategy to inhibit the phosphoryl-transfer reaction (Nguyen and Venditti 2020).

Despite the wealth of knowledge we possess about the coupling between EIC conformational flexibility and enzymatic activity, very little is known about if and how EIN conformational dynamics impact the function of the enzyme. Indeed, while a comparison of the experimental atomic-resolution structures of EI indicates that the open-to-close conformational change is coupled to a rigid body reorientation of the α subdomain relative to the α/β subdomain of EIN (commonly referred to as state A-to-state B equilibrium, Figure 1B) and that protein phosphorylation shifts the χ2 angle of His^189^ from the *g* ^*+*^ to *g* ^*−*^ rotameric state (Figure 1C), it is not clear if these conformational equilibria are active in isolated EIN and if their external perturbation can impact turnover. Addressing these questions will advance our understanding of how synergistic couplings among intradomain, interdomain, and intersubunit conformational equilibria affect the function of a multidomain oligomeric protein such as EI, and will provide new perspectives toward the development of EI inhibitors that act on the EIN domain.

Here we investigate the structure and dynamics of isolated EIN in its native and phosphorylated forms by solution NMR spectroscopy. While we do not detect evidence of an active state A/state B equilibrium, relaxation dispersion experiments indicate that the conformational exchange between the *g* ^*+*^ and *g* ^*−*^ rotameric states of His^189^ is active in the isolated EIN and modulated by protein phosphorylation. Furthermore, we engineered EIN constructs with modulated thermostability and conformational flexibility by hybridizing EIN from mesophilic and thermophilic organisms. Biophysical characterization of the wild-type and hybrid EIN constructs indicates that the rotameric equilibrium is slower in the thermophilic enzyme than in the mesophilic protein and that a single serine to alanine mutation is responsible for the increased activation energy in the thermophilic species. Finally, we performed functional characterization of the wild-type mesophilic and thermophilic EI, as well as of EI constructs that incorporate the hybridizing mutations. Our data show that, although the His^189^ rotameric equilibrium is required for the correct functioning of EI, the rate of the phosphoryl transfer reaction is independent on the kinetics of the conformational change, indicating that the *g* ^*+*^-to-*g* ^*−*^ transition is not the rate limiting step for catalysis.

## Materials and Methods

### Protein Expression and Purification

*E. coli and T. tengcongensis* EI, EIN, and HPr were expressed and purified as previously reported (Suh et al., 2008; Venditti and Clore, 2012; Dotas and Venditti, 2019). Single point mutations were introduced using the QuikChange site-directed mutagenesis.

### Thermal Stability and Circular Dichroism

Thermal-induced unfolding of EIN was investigated in a 1 mm, 400 μl, quartz cuvette sample cell using a Jasco J-710 spectropolarimeter. Samples were prepared in H_2_O at a protein concentration of ∼0.5 mg/ml. Ellipticity (θ_222nm_) at the 222 nm wavelength was monitored over a 1°C/min temperature gradient ranging from 35 to 75°C and 65–95°C for mesophilic and thermophilic EIN, respectively. The melting temperature (*T*_*m*_) was calculated as the maximum value of the derivative of θ_222nm_ with respect to temperature.

### Nuclear Magnetic Resonance Spectroscopy

NMR samples were prepared in 20 mM Tris-HCl buffer (pH 7.4), 100 mM NaCl, 4 mM MgCl_2_, 1 mM ethylenediaminetetraacetic acid (EDTA), 2 mM dithiothreitol (DTT), and 90% H_2_O/10% D_2_O (v/v). For protein phosphorylation, samples were incubated for 1 h at 37°C with <1 μM of mesophilic EI (*e*EI) or thermophilic EI (*t*EI), <1 μM of mesophilic HPr (*e*HPr) or thermophilic HPr (*t*HPr), and ∼30 mM of PEP prior to acquisition. Completion of phosphorylation reactions were confirmed by the disappearance of the NMR peaks of the unphosphorylated species from the ^1^H-^15^N TROSY spectrum of the proteins. The protein concentration, in subunits, was ∼1 mM for all NMR experiments, unless stated otherwise.

NMR spectra were acquired on Bruker 800, 700, and 600 MHz spectrometers equipped with z-shielded gradient triple resonance cryoprobes. Spectra were processed using NMRPipe (Delaglio et al., 1995) and analyzed using NMRFAM-SPARKY (Lee et al., 2015). ^1^H-^15^N TROSY (transverse relaxation-optimized spectroscopy) (Pervushin et al., 1998) and methyl-TROSY (Tugarinov et al., 2003) experiments were acquired using previously described pulse schemes. Resonance assignments of the ^1^H-^15^N TROSY spectra for *e*EIN and *t*EIN were transferred from previous reports (BMRB accession codes 4,106 and 27,833, respectively) (Garrett et al., 1999; Dotas and Venditti, 2019). Resonance assignments of the ^1^H-^15^N TROSY spectra for phosphorylated *e*EIN (*e*EIN-P) was kindly provided by Drs. Clore and Suh (Suh et al., 2008). Sequential ^1^H/^15^N/^13^C backbone assignments of phosphorylated *t*EIN (*t*EIN-P) were achieved using TROSY versions of conventional 3D triple resonance correlation experiments [HNCO, HNCA, HNCACB, HN(CO)CA, and HN(CO)CACB] (Clore and Gronenborn, 1998). Assignment of the ^1^H-^13^C_methyl_ correlations of *t*EIN-P was performed using out and back experiments (Tugarinov et al., 2014). NMR resonance assignments for *t*EIN-P were deposited on the BioMagResBank (Ulrich et al., 2008) (accession code 50386).

The weighted combined ^1^H/^15^N chemical shift perturbations (∆_H/N_) resulting on the ^1^H-^15^N TROSY spectra of EIN from phosphorylation of His^189^ were calculated using the following equation (Mulder et al., 1999):

$$\Delta_{H/N}\;=\sqrt{{\left(\Delta \delta_{H}W_{H}\right)}^{2}+{\left(\Delta \delta_{N}W_{N}\right)}^{2}}$$ (1)

where *W*_*H*_ (=1) and *W*_*N*_ (=0.154) are the weighting factors for the ^1^H and ^15^N amide chemical shifts and ∆δ_*H*_ and ∆δ_*N*_ symbolize the ^1^H and ^15^N chemical shift differences in ppm between the unphosphorylated and phosphorylated states.

^15^N-*R*_1_ and *R*_1ρ_ experiments were recorded at 40°C and a ^1^H frequency of 800 MHz, utilizing heat-compensating pulse schemes with a TROSY readout (Lakomek et al., 2012). A recycle delay of 1.5 s was used for both *R*_1_ and *R*_1ρ_ experiments, with the spin-lock field (*ω*_1_) for the *R*_1ρ_ experiments set to 1 kHz. Relaxation delay durations were 0, 120, 280, 440, 640, 800, 1,040, and 1,200 ms for *R*_1_ and 0.2, 4.2, 7.2, 15, 23.4, 32.4, 42, 52.2, and 60 ms for *R*_1ρ_, respectively. *R*_1_ and *R*_1ρ_ values were determined by fitting time-dependent exponential restoration of peak intensities at increasing relaxation delays. *R*_*2*_ values were extracted from the measured *R*_*1*_ and *R*_1ρ_ values. Global rotational correlation times (τ_c_) were estimated from the mean *R*_1_ and *R*_2_ values, excluding residues displaying enhanced local dynamics on the ps-ns timescale, using the following equation (Kay et al., 1989):

$$\tau_{c}\approx \;\frac{1}{4\pi \nu_{N}}\sqrt{6\frac{R_{2}}{R_{1}}-7}\;$$ (2)

where $\nu_{N}$ is the ^15^N resonance frequency in Hz, and *R*_1_ and *R*_2_ are the average determined values of the ^15^N relaxation rates.

^15^N and ^13^C_methyl_ relaxation dispersion (RD) experiments were conducted at 5, 10, 15, and 20°C using a well-established protocol (Singh et al., 2021). In brief, a pulse sequence that measures the exchange contribution for the TROSY component of the ^15^N magnetization (Loria et al., 1999) and a pulse scheme for ^13^C single-quantum CPMG (Carr-Purcell-Meinboom-Gill) RD described by Kay and co-workers (Lundström et al., 2007) were employed. Off-resonance effects and pulse imperfections were minimized using a four-pulse phase scheme (Yip and Zuiderweg, 2004). CPMG RD experiments were performed at a ^1^H frequency of 800 and 600 MHz with fixed relaxation delays (*T*_*relax*_) of 60 and 30 ms, for ^15^N and ^13^C_methyl_ experiments, respectively. Different numbers of refocusing pulses were implemented to produce effective CPMG fields (ν_*CPMG*_) varying from 50 to 1,000 Hz (Mulder et al., 2001). Experimental errors on the transverse relaxation rates (*R*_2_) were estimated from the noise level estimated with the NMRFAM-SPARKY software. The resulting RD curves acquired at multiple temperatures and magnetic fields were globally fit to a two-site exchange model using the Carver-Richard equation (Carver and Richards, 1972), as described by Dotas et al. (2020).

Backbone amide ^1^D_NH_ residual dipolar couplings (RDCs) were measured at 40°C by taking the difference in ^1^J_NH_ scalar couplings in isotropic and alignment media. Phage pf1 (16 mg/ml; ASLA Biotech) was the employed alignment media and the ^1^J_NH_ couplings were measured using the RDCs by TROSY pulse scheme (Fitzkee and Bax, 2010). Xplor-NIH (Schwieters et al., 2003) was used to compute singular value decomposition (SVD) analysis of the RDC values.

### Enzyme Kinetic Assays

*e*EI, *e*EI_S191A_, *t*EI, and *t*EI_A191S_ were investigated for their ability to catalyze the transfer of the phosphoryl group from PEP to HPr. Assays were performed on a Bruker 700 MHz spectrometer at 25°C using ^1^H-^15^N Selective Optimized Flip Angle Short Transient (SOFAST) NMR experiments (Schanda et al., 2005), using a protocol previously described (Nguyen et al., 2018). The reaction buffer was 20 mM Tris-HCl buffer (pH 7.4), 100 mM NaCl, 4 mM MgCl_2_, 1 mM ethylenediaminetetraacetic acid (EDTA), 2 mM dithiothreitol (DTT), and 90% H_2_O/10% D_2_O (v/v). The reaction volume was 500 μl. All enzymatic assays were run at fixed concentrations of enzyme (∼0.005 μM), HPr (600 μM), and PEP (1 mM). The initial velocities (ν_*0*_) for the phosphoryl transfer reactions were determined by plotting the concentration of unphosphorylated HPr determined by the ^1^H-^15^N cross-peak intensities, as described by Nguyen et al. (2018) vs. time (Figure 5D). All assays were performed in triplicates to estimate the experimental error.

### Mass Spectrometry

A binary ACQUITY UPLC H-Class system coupled with Synapt G2-Si HDMS system (Waters, Milford, MA) and electrospray ionization (ESI) source was used to determine the intact masses of phosphorylated and non-phosphorylated *t*EIN. Starting samples were prepared by diluting 10 μl of the NMR samples of phosphorylated and non-phosphorylated *t*EIN with HPLC grade H_2_O to final concentration of 2 µM. 1 µl of each sample was injected in the mass spectrometer.

UPLC separations were performed using a Restek Ultra C4 column (5 um 50 mm × 1 mm) with a flow rate of 0.4 ml/min. Solvents used were 0.1% formic acid in HPLC grade H_2_O (solvent A) and 0.1% formic acid in acetonitrile (solvent B, mobile phase). The gradient used started with an initial condition of 5% B for 1 min, followed by a 7 min gradient of 5–100% solvent B. This was held for 4 min before dropping back to the initial 5% buffer B in 1 min and held for the remainder of the run (total 20 min).

The eluant from the UPLC was introduced to the Waters Synapt G2-Si HDMS with TOF mass analyzer using a Waters Lockspray Source (300–5,000 Da mass range). Finally, the intact mass was determined by deconvolution of mass spectra using the MassLynx 4.2 software.

## Results

In this contribution, we investigate the structure and conformational dynamics of native and phosphorylated EIN from two organisms: a mesophilic bacterium (*Escherichia coli*) and a thermophilic organism (*Thermoanaerobacter tengcongensis*). The two proteins are referred to as *e*EIN and *t*EIN in the unphosphorylated state, and *e*EIN-P and *t*EIN-P in the phosphorylated state throughout the manuscript, respectively. Further, we examined the effect of two single-point mutations, Ser^191^→Ala^191^ within *e*EIN and Ala^191^→ Ser^191^ within *t*EIN, on protein structure and dynamics. These mutants are denoted *e*EIN_S191A_ and *t*EIN_A191S_, respectively. Similar notations are used for HPr and the full-length EI (*e*HPr, *t*HPr, *e*EI, *t*EI, *e*EI_S191A_, and *t*EI_A191S_). The full-length *e*EI and *t*EI share similar sequence (overall identity = 54% and active site identity = 100%) (Supplementary Figure 1) and 3D structure (Oberholzer et al., 2005; Teplyakov et al., 2006; Navdaeva et al., 2011; Evangelidis et al., 2018), but have been shown to be optimally active at 37 and 65°C, respectively, (Navdaeva et al., 2011). The sequence identity of isolated *e*EIN and *t*EIN is 48% (Supplementary Figure 1).

### Effect of Phosphorylation on the Structure of EIN

Previous structural investigations on *e*EIN have shown that phosphorylation does not affect the conformation of the *e*EIN backbone, but results in a transition of the χ2 angle of His^189^ from the *g* ^*+*^ to the *g* ^*−*^ rotameric state. Such conformational change breaks the hydrogen bond between the Nε2 atom of His^189^ and the hydroxyl group of Thr^168^ and grants the inbound phosphoryl group access to the Nε2 atom (Figure 1C) (Garrett et al., 1999; Suh et al., 2008).

**FIGURE 2:**
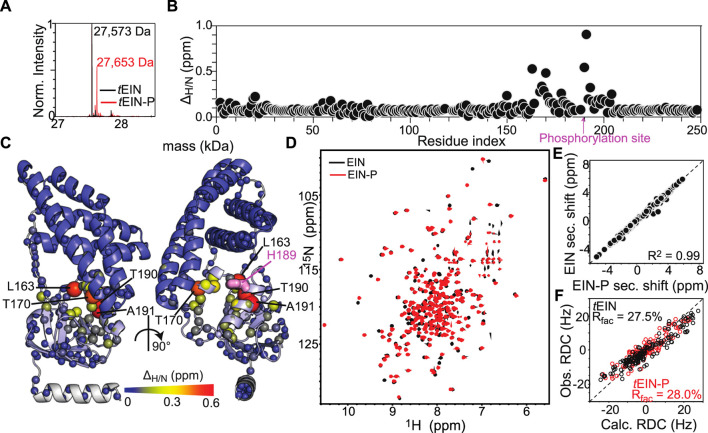
Phosphorylation induced structural perturbations in *t*EIN. **(A)** Deconvoluted masses of *t*EIN (black) and *t*EIN-P (red) from LC-MS. **(B)** Weighted combined chemical shift perturbations (*Δ*_*H/N*_) induced by phosphorylation of *t*EIN on the ^1^H-^15^N TROSY spectra of the protein are plotted against residue index. **(C)** The experimental *Δ*_*H/N*_ values are plotted on the structure of *t*EIN (PDB code: 5WOY). Two different *t*EIN orientations are shown. The relationship between sphere size and color and the value of *Δ*_*H/N*_ is depicted by the color bar. The His^189^ side chain is shown as pink sticks. **(D)** 800 MHz ^1^H-^15^N TROSY spectra of *t*EIN (black) and *t*EIN-P (red). **(E)** Correlation between the secondary Cα chemical shifts measured for *t*EIN (*y*-axis) and *t*EIN-P (*x*-axis). **(F)** SVD fitting of the ^1^D_NH_ RDC data measured for *t*EIN (black) and *t*EIN-P (red) against the solution structure of *t*EIN (PDB code: 5WOY).

Here, the effect of phosphorylation at the His^189^ position on the structure of *t*EIN has been evaluated by NMR chemical shift perturbations and backbone amide residual dipolar coupling (^1^D_NH_ RDC) data. *t*EIN-P samples were prepared by adding catalytic amounts (<1 µM) of *t*EI and *t*HPr, and a large excess (∼30 mM) of PEP directly to an NMR tube containing ∼1 mM of ^15^N-labeled *t*EIN. The phosphoryl transfer reaction is slow on the chemical shift time scale, and distinct NMR peaks are observed for *t*EIN and *t*EIN-P (Figure 2D). Therefore, completion of the phosphorylation reaction was determined by disappearance of the NMR peaks of the unphosphorylated species from the ^1^H-^15^N TROSY spectrum of the protein. In addition, small aliquots (∼10 µl) of the NMR sample were taken before and after addition of EI, and analyzed by Liquid Chromatography with tandem mass spectrometry (LC-MS-MS). The data indicate that the *t*EIN mass increases by 80 Da upon incubation with *t*EI, *t*HPr, and PEP (Figure 2A), which is consistent with the addition of a single phosphoryl group.

NMR resonance assignments for *t*EIN have been previously reported (BMRB code: 27833) (Dotas and Venditti, 2019). Assignments for *t*EIN-P were performed using conventional triple resonance correlation experiments (see “Materials and Methods”) and deposited in the BioMagResBank (BMRB code: 50386). ^1^H/^15^N chemical shift perturbations (Δ_*H/N*_) induced by phosphorylation in the ^1^H-^15^N TROSY spectrum of *t*EIN are plotted against residue index in Figure 2B and displayed as a gradient on the protein structure in Figure 2C. Significant (>0.3 ppm) Δ_*H/N*_ values are observed exclusively in the vicinity of the phosphorylation site on the α/β subdomain and are presumably a result of electronic effects that arise from the presence of the phosphoryl group as well as ring current effects from the change in the χ2 angle of His^189^ to accommodate the phosphoryl group at the Nε2 position. Such hypothesis is supported by the excellent agreement between the secondary Cα chemical shifts measured for phosphorylated and unphosphorylated *t*EIN (Figure 2E), which demonstrates that no transition in backbone conformation occurs upon phosphorylation. Further insight into the effect of phosphorylation on the structure of EIN was obtained by the analysis of ^1^D_NH_ RDC data measured for *t*EIN and *t*EIN-P aligned in a dilute liquid crystalline medium of phage pf1. RDCs of fixed bond vectors, such as the backbone N–H bond vector, are dependent on the orientation of the bond vectors relative to the alignment tensor and thus provide a very sensitive indicator of changes in relative domain orientations (Tjandra and Bax, 1997; Venditti et al., 2016). Singular value decomposition (SVD) fitting of the experimental RDCs measured for *t*EIN and *t*EIN-P to the coordinates of the solution structure of unphosphorylated *t*EIN (PDB code: 5WOY) (Evangelidis et al., 2018) yields R-factors (Clore and Garrett, 1999) of 27.5 and 28.0%, respectively, indicating good agreement between experimental and back-calculated data (Figure 2F). Consequently, one can conclude that the relative orientation of the α and α/β subdomains remains unperturbed by phosphorylation.

In summary, consistent with structural investigations performed previously on *e*EIN (Suh et al., 2008), the solution NMR data presented in this section indicate that phosphorylation of *t*EIN results in a localized transition of the rotameric state of the His^189^ side chain and does not propagate into larger conformational rearrangements that involve the backbone of the protein.

### Effect of Phosphorylation on the ps-ns Dynamics

**FIGURE 3:**
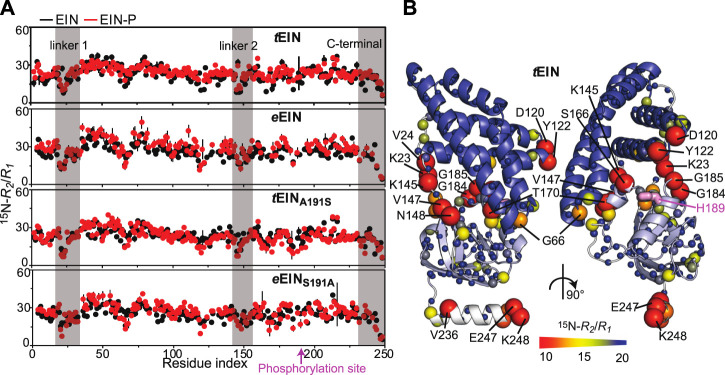
Effect of phosphorylation on the ps-ns dynamics of EIN. **(A)** ^15^N-*R*_*2*_/*R*_*1*_ data measured at 800 MHz and 40°C for *t*EIN (top), *e*EIN (second from top), *t*EIN_A191S_ (second from bottom), and *e*EIN_S191A_ (bottom) are plotted vs. the residue index. The unphosphorylated and phosphorylated states are colored black and red, respectively. The localization of linker 1, linker 2, and the C-terminal end are indicated by transparent gray boxes. **(B)** The ^15^N-*R*_*2*_/*R*_*1*_ data measured for *t*EIN are displayed as a gradient on the solution structure of the protein (PDB code: 5WOY) according to the color bar. The His^189^ side chain is shown as pink sticks.

ps-ns timescale dynamics were investigated for *e*EIN, *e*EIN-P, *t*EIN, and *t*EIN-P using NMR relaxation experiments. NMR samples of *e*EIN-P were produced as described previously (Suh et al., 2008). Residue-specific ^15^N-*R*_*1*_ and ^15^N-*R*_*2*_ values were obtained at 800 MHz and 40°C by acquisition of TROSY-detected *R*_*1*_ and *R*_*1ρ*_ experiments (Lakomek et al., 2012) on uniformly ^15^N-labeled protein. ^15^N-*R*_*2*_/*R*_*1*_ ratios are graphed as a function of residue index in Figure 3A and depicted as a gradient on the solution structure of *t*EIN in Figure 3B. For globular diamagnetic proteins, global tumbling is the only significant contribution to ^15^N relaxation and the *R*_*2*_/*R*_*1*_ values are expected to be constant throughout the amino acid sequence and proportional to the rotational correlation time (τ_c_) (Kay et al., 1989). Therefore, residues that produce lower than average *R*_*2*_/*R*_*1*_ values likely undergo additional local motion on the ps-ns timescale that decrease the effective correlation time experienced by the N-H bond. Analysis of the NMR relaxation data in Figure 3A indicates that *e*EIN, *e*EIN-P, *t*EIN, and *t*EIN-P tumble with a τ_c_ ∼ 11 ns, which is consistent with the theoretical τ_c_ calculated for a globular protein of the EIN size (∼11 ns). Notably, several regions of the protein exhibit lower than average *R*_*2*_/*R*_*1*_ values, suggesting the presence of local backbone dynamics on the ps-ns timescale. These regions cluster into the unstructured linkers connecting the α and α/β subdomains (linker 1: residues 19–32; linker 2: residues 143–156) and at the C-terminal end of EIN (Figure 3B). However, phosphorylation of His^189^ elicits no observable change in the distribution of the *R*_*2*_/*R*_*1*_ values, indicating that the phosphoryl transfer reaction does not affect the ps-ns timescale dynamics of EIN (Figure 3A).

### Effect of Phosphorylation on the μs-ms Dynamics

μs-ms timescale dynamics in *e*EIN, *e*EIN-P, *t*EIN, and *t*EIN-P were investigated by ^15^N and ^13^C_methyl_ Carr-Purcell-Meiboom-Gill (CPMG) relaxation dispersion (RD) spectroscopy (Mittermaier and Kay 2006). Experiments were acquired on U-(^2^H,^15^N)/Ile (d_1_)-^13^CH_3_/Val, Leu-(^13^CH_3_/^12^C_2_H_3_)-labeled samples at two different static fields (600 and 800 MHz) and four different temperatures (5, 10, 15, and 20°C). Simultaneous investigation of RD profiles measured at multiple temperatures returns a comprehensive characterization of the kinetics and thermodynamics of conformational exchange processes between species with distinct chemical shifts occurring on a timescale ranging from ∼0.1 to ∼10 ms, by providing enthalpy (*ΔH*), entropy (*ΔS*), activation enthalpy (*Δ* ^*‡*^ *H),* and activation entropy (*Δ* ^*‡*^ *S*) for the conformational equilibrium (Purslow et al., 2018).

**FIGURE 4:**
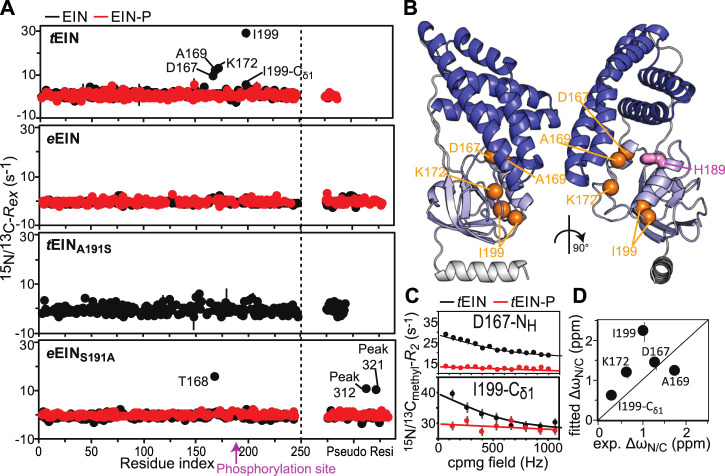
Effect of phosphorylation on the μs-ms dynamics for EIN. **(A)** ^15^N and ^13^C_methyl_ exchange contributions to the transverse relaxation rate (*R*_*ex*_) measured at 10°C and 800 MHz are graphed vs. residue index for *t*EIN (top), *e*EIN (second from top), *t*EIN_A191S_ (second from bottom), and eEIN_S191A_ (bottom). The unphosphorylated and phosphorylated states are colored black and red, respectively. Unassigned cross-peaks were given arbitrary pseudo residue indexes larger than 300. **(B)** The NMR correlation showing *R*_*ex*_ values larger than 5 s^−1^ at 10°C and 800 MHz for *t*EIN are displayed as orange spheres on the solution structure of *t*EIN (PDB code: 5WOY). The His^189^ side chain is shown as pink sticks. **(C)** Examples of typical RD profiles measured at 10°C and 800 MHz for unphosphorylated (black) and phosphorylated (red) *t*EIN. Data are shown for the Asp^167^ NH (top) and the Ile^199^ δ1-methyl (bottom) groups. The experimental data are shown as circles. The best fit curves are shown as lines. The complete set of analyzed data are shown in Supplementary Figure 2. **(D)** The peak specific ∆ω_N_ and ∆ω_C_ parameters obtained from fitting the RD profiles of *t*EIN are plotted vs. the change in ^15^N and ^13^C_methyl_ chemical shift induced by protein phosphorylation. The poor agreement between experimental and fitted ∆ω_N_ observed for Ile^199^ might be due to the fact that the phosphoryl group is oriented toward this amide group in the phosphorylated enzyme (Figure 1C). Because the RD experiments were acquired on unphosphorylated EIN, the effects on the ^15^N chemical shift of the electrostatic interactions between the phosphoryl group and the Ile^199^ backbone amide are not observable in the fitted ∆ω_N_.

Exchange contributions toward the transverse relaxation rates (*R*_*ex*_) are plotted against residue index in Figure 4A. Large (>5 s^−1^) *R*_*ex*_ values were detected for amino acids that cluster in the vicinity of the active site His^189^ on the α/β subdomain of *t*EIN (Figures 4A,B). In particular, Asp^167^, Ala^169^, Lys^172^ localize on the partially structured helix that is in direct contact with His^189^, while Ile^199^ is located at the C-terminal end of the short helix that comprises the phosphorylation site. Contrarily, *e*EIN revealed no significant *R*_*ex*_ values (Figure 4A), suggesting the observed dynamics in *t*EIN may be too fast to be detected by CPMG within its mesophilic analogue. Interestingly, all the ^15^N and ^13^C_methyl_ RD curves measured at multiple temperatures and static magnetic fields for Asp^167^, Ala^169^, Lys^172^, and Ile^199^ within *t*EIN could be fit simultaneously to a model describing the interconversion between two conformational states (Figure 4C; Supplementary Figure 2). In this global fitting procedure, the activation (*Δ* ^*‡*^ *G)* and standard (*ΔG)* free energy of the conformational equilibrium were optimized as global parameters, whereas the ^15^N and ^13^C chemical shift differences between the two conformational states (*Δω*_*N*_ and *Δω*_*C*_, respectively) were treated as peak-specific and temperature independent parameters. The exchange rate (*k*_*ex*_) and the fractional population of the minor conformational state (*p*_*b*_) were calculated at each temperature from the fitted values of *Δ* ^*‡*^ *G* and *ΔG* using the general form of the Eyring and reaction isotherm equations, respectively. This fitting procedure reduces the number of optimized parameters from 28 (*k*_*e*x_, *p*_*b*_, and *Δω* for five NMR peaks at four experimental temperatures) to 7 (*Δ* ^*‡*^ *G*, *ΔG*, and *Δω* for five NMR peaks), and it is justified if the heat capacity of activation remains constant over the experimental temperature range (5–20°C) (Nguyen et al., 2017). For completeness, it should be noted that the intrinsic ^15^N and ^13^C_methyl_ transverse relaxation rates were also optimized as peak-specific parameters, therefore increasing the overall number of fitted parameters. Also, in order to improve convergence of the fitting algorithm, the *Δω*_*N*_ parameters were restrained to be larger than 1 ppm. Recently we have used a similar fitting protocol to model the temperature dependence of the μs-ms dynamics in the EIC domain of the enzyme (Dotas et al., 2020).

A summary of the optimized parameters is reported in Table 1. Examples of the global fit are provided in Figure 4C. Curves for all the analyzed RD profiles are provided in Supplementary Figure 2. The optimized *Δ* ^*‡*^ *G* is 50,228 ± 227J mol^−1^, which translates into exchange rate constants (sum of forward and backward rate constants, *k*_*ab*_ and *k*_*ba*_, respectively) of 2,117 ± 236, 3,162 ± 347, 4,660 ± 503, and 6,780 ± 719 s^−1^ at 5, 10, 15, and 20°C, respectively. The optimized *ΔG* is 5,601 ± 330 J mol^−1^, resulting in *p*_*b*_ values of 8.0 ± 0.4, 8.4 ± 0.4, 8.7 ± 0.4, and 9.0 ± 0.4% at 5, 10, 15, and 20°C, respectively (Table 1). *k*_*ab*_ and *k*_*ba*_ were calculated from the values of *k*_*ex*_ and *p*_*b*_, and their temperature dependence was modeled using the van’t Hoff and Eyring equations to obtain *ΔH*, *ΔS*, *Δ* ^*‡*^ *H*, and *Δ* ^*‡*^ *S* of the *t*EIN conformational equilibrium (Table 1).

**TABLE 1:** Kinetic and thermodynamic parameters for the µs-ms dynamics detected by CPMG.

	*k*_*ab*_/*k*_*ba*_ (s^−1^)^a^				*Δ* ^*‡*^ *H*_*ab*_/*Δ* ^*‡*^ *H* ^b^ ^,^ ^a^(kJ mol^−1^)^b^	*Δ* ^*‡*^ *S*_*ab*_/*Δ* ^*‡*^ *S* ^b^ ^,^ ^a^(J K^−1^ mol^−1^)^b^	*p* ^b^(%)^c^				*ΔH*	*ΔS*
	5°C	10°C	15°C	20°C			5°C	10°C	15°C	20°C	(kJ mol^−1^)^d^	(J K^−1^ mol^−1^)^d^
*t*EIN	171	265	406	613	55 ± 1	−2 ± 1	8.1	8.4	8.7	9.0	6 ± 3	0 ± 39
	1946	2,897	4,255	6,168	50 ± 1	−2 ± 1						
*e*EIN_S191A_	361	554	837	1,249	54 ± 1	−2 ± 1	8.9	9.3	9.6	10.0	5 ± 3	−2 ± 22
	3,686	5,425	7,879	11,297	48 ± 1	−3 ± 1						

a The major and minor states of the equilibrium are referred to as *a* and *b*, respectively. *k*_*ab*_ and *k*_*ba*_ are the rate constants for the transition from *a* to *b* and from *b* to *a*, respectively, and are calculated from the values of the optimized parameters *k*_*ex*_ (= *k*_*ab*_ + *k*_*ba*_) and *p*_*b*_. The upper and lower numbers refer to *k*_*ab*_ and *k*_*ba*_, respectively. Errors on rate constants are < 15% of the reported values.

b Activation enthalpies and entropies for the *a* to *b* and *b* to *a* transitions were calculated by fitting the temperature dependence of *k*_*ab*_ and *k*_*ba*_ to the Eyring equation, respectively. The upper and lower numbers refer to *k*_*ab*_ and *k*_*ba*_, respectively.

c Errors on populations are <5% of the reported values.

d Enthalpy and entropy changes associated with the conformational equilibrium were calculated by using the van’t Hoff equation. The equilibrium constant (*K*_*eq*_) at each temperature was calculated using the formula *K*_*eq*_ = *p*_*b*_/(1−*p*_*b*_).

From the analysis of the kinetic, thermodynamic, and NMR parameters obtained by the RD study at multiple temperatures it is apparent that 1) *t*EIN is in equilibrium between two conformational states, 2) the relative thermodynamic stability of the two states is dictated by enthalpic contributions to the free energy (Table 1), and 3) the ^15^N and ^13^C chemical shift differences between the two conformational states correlates with the change in ^15^N and ^13^C chemical shift (*Δ*_*N*_ and *Δ*_*C*_, respectively) induced by phosphorylation of *t*EIN (Figure 4D). These findings suggest that the μs-ms dynamics detected in *t*EIN by RD experiments report on the equilibrium between the *g* ^*+*^ and *g* ^*−*^ rotameric states of His^189^ that breaks the hydrogen bond between the Thr^168^ and His^189^ side-chains and makes the Nε2 atom accessible to the incoming phosphoryl group. Consistent with this hypothesis, the fitted value for Δ*H* (6 kJ mol^−1^) is comparable with the reported energies for the weak intramolecular hydrogen bonds involving the hydroxyl group on Ser or Thr side-chains (∼7 kJ mol^−1^) (Pace et al., 2014a; Pace et al., 2014b).

To further test this model, the effect of phosphorylation on the μs-ms dynamics of *t*EIN was investigated at 10°C by acquisition of RD experiments on *t*EIN-P. *t*EIN-P was prepared enzymatically as described above. However, in this case, *t*EI, *t*HPr, and excess PEP were purified out of the final NMR sample by anion exchange chromatography. This additional purification step is required to remove any possible complex between *t*EIN and other molecules in the sample that, even at very dilute concentrations (<1% of the total *t*EIN concentration), might cause artifacts in the observed RD profiles (Singh et al., 2021). Since phosphorylated histidine is a labile post-translational modification that decays over the time, the phosphorylation state of the purified *t*EIN-P sample was checked by ^1^H-^15^N TROSY before and after acquisition of the RD experiments at 10°C. Both NMR spectra show no sign of unphosphorylated *t*EIN cross-peaks, confirming that *t*EIN remained fully phosphorylated during NMR data acquisition.

As expected, the RD data measured for *t*EIN-P at 800 MHz shows that phosphorylation completely suppresses μs-ms dynamics within *t*EIN (Figures 4A,C). Indeed, addition of the bulky phosphoryl group at the Nε2 position hampers formation of the Thr^168^-His^189^ hydrogen bond and locks the side-chain of His^189^ in the *g* ^−^ rotameric state (Figure 1C). It is worth reinstating that no *R*_*ex*_ value larger than 5 s^−1^ was measured for *e*EIN and, as expected, phosphorylation of *e*EIN resulted in no observable change in the *R*_*ex*_ distribution (Figure 4A). Being that the experimental structures of phosphorylated and unphosphorylated *e*EIN indicate that the protein must undergo the His^189^ rotameric transition to accommodate the phosphoryl group (Figure 1C), our data suggests the *g* ^*+*^/*g* ^−^ exchange in *e*EIN is faster than in *t*EIN and, therefore, not detected by RD experiments.

### Engineering eEIN/tEIN Hybrids With Modulated Conformational Dynamics

**FIGURE 5:**
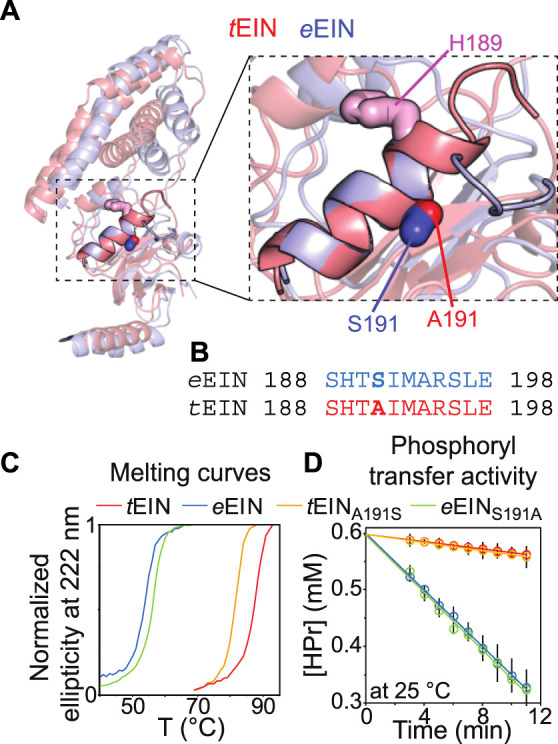
Design and characterization of *e*EIN/*t*EIN hybrids. **(A)** Structure alignment of α-helix 6 in the solution structure of *e*EIN (PDB code: 2EZA) (blue) and *t*EIN (PDB code: 5WOY) (red). The side chain of His^189^ is shown as pink sticks. The side chain of Ser^191^ in *e*EIN is shown as blue sticks. The side chain of Ala^191^ in *t*EIN is shown as red sticks. **(B)** Sequence alignment of α-helix 6 in *e*EIN (blue) and *t*EIN (red). Full sequence alignment of the mesophilic and thermophilic protein is provided in Supplementary Figure 1. **(C)** Temperature-induced unfolding of *e*EIN (blue), *e*EIN_S191A_ (green), *t*EIN_A191S_ (orange), and *t*EIN (red). **(D)** Rate of HPr phosphorylation catalyzed by 0.005 μM *e*EI (blue), *e*EI_S191A_ (green), *t*EI_A191S_ (orange), and *t*EI (red) in the presence of 1 mM PEP at 25°C. The phosphorylation rate (V_0_) was obtained by linear fitting (solid lines) of the decrease in concentration of unphosphorylated HPr (open circles) vs. time. A V_0_ of ∼25 μM/min was obtained for *e*EI and *e*EI_S191A_. A V_0_ of ∼3 μM/min was obtained for *t*EI and *t*EI_A191S_.

We have recently shown that hybridizing proteins from mesophilic and thermophilic bacteria is an effective strategy to produce active enzymes with modulated conformational dynamics and biological function (Dotas et al., 2020). Indeed, by merging the scaffold of EIC from *T. tengcongensis* with the active site loops of the *E. coli* enzyme we engineered a hybrid EIC variant that displays the thermal stability of the thermophilic protein and the high active site flexibility and low-temperature activity of the mesophilic enzyme. In contrast, implanting the active site loops from *T. tengcongensis* EIC onto the scaffold of *E. coli* EIC resulted in a construct that is more rigid and less active than the mesophilic enzyme (Dotas et al., 2020). Here, *e*EIN/*t*EIN hybrids are engineered to investigate the relationship between the kinetics of the His^189^ rotameric equilibrium and turnover number. Comparison of the experimental atomic-resolution structures shows that the N-terminal end of α-helix 6 (which comprises the His^189^) in *t*EIN is two residues longer than in *e*EIN (Figure 5A). Alignment of the amino acid sequences reveals a single Ser^191^Ala mutation within α-helix 6 moving from the mesophilic to the thermophilic construct (Figure 5B; Supplementary Figure 1). As alanine residues are known to promote helix formation in proteins (Panja et al., 2015), we hypothesize that the Ser^191^Ala mutation provides structural stabilization to α-helix six and is responsible for the slower rotameric equilibrium observed for His^189^ in *t*EIN. To test this hypothesis, we investigated the structure, dynamics, and thermal stability of *e*EIN_S191A_ and *t*EIN_A191S_ by solution NMR and circular dichroism (CD).

Although a comparison of the ^1^H-^15^N TROSY spectra measured for the wild type and mutant proteins shows that mutations at the 191 position provides minimal perturbations to the NMR spectra and, therefore, to the solution fold of EIN (Supplementary Figure 3), the temperature-induced unfolding data acquired by CD reveal that the introduced mutations result in sizable and opposing effects on the thermostability of *e*EIN and *t*EIN (Figure 5C). In particular, melting temperatures (*T*_*m*_) of 54.0, 56.5, 82.0, and 88.0°C were determined for *e*EIN, *e*EIN_S191A_, *t*EIN_A191S_, and *t*EIN from the first derivative of the unfolding sigmoidal curves, respectively. This pattern of *T*_*m*_ values confirms that introducing an Ala residue at position 191 increases the thermal stability of *e*EIN, while the Ala^191^Ser mutation results in destabilization of *t*EIN.

Analysis of the ^15^N-*R*_*2*_/*R*_*1*_ vs. residue plots reveals a similar pattern for the wild type and mutant proteins, indicating that the introduced mutations do not affect the ps-ns dynamics in native and phosphorylated EIN (Figure 3A). On the other hand, mutations of residue 191 generate observable changes in the μs-ms timescale dynamics of the protein. Indeed, no NMR peak with *R*_*ex*_ > 5 s^−1^ is detected in the NMR spectra of *t*EIN_A191S_ (Figure 4A), which is consistent with the hypothesis that Ala^191^Ser mutation in *t*EIN speeds up the rotameric equilibrium of His^189^. In contrast, the Ser^191^Ala mutation in *e*EIN introduces *R*_*ex*_ > 5 s^−1^ at three ^1^H-^15^N TROSY correlations. Although we were able to confidently assign only one of these three NMR signals, we ascribe the appearance of exchange induced effects on the spectra of *e*EIN_S191A_ to the rotameric equilibrium of His^189^ for the following reasons: 1) The assigned NMR correlation with *R*_*ex*_ > 5 s^−1^ (Thr^168^) localizes in the same region observed to experience exchange contributions to *R*_*2*_ in *t*EIN (Figure 4A), 2) Global fitting of the RD profiles acquired for the three NMR signals at multiple temperatures and static fields (Supplementary Figure 4) produces kinetic and thermodynamic parameters that are similar to the ones obtained for the His^189^ rotameric equilibrium in *t*EIN (Table 1), and 3) Phosphorylation of *e*EIN_S191A_ results in a complete quenching of μs-ms dynamics (Figure 4A).

Overall, the NMR and CD data reported above support the hypothesis that the identity of the residue at position 191 controls helix 6 stability and the dynamics of the *g* ^*+*^/*g* ^*−*^ equilibrium of His^189^. To test the dependency of the EI biological function on the kinetics of the His^189^ rotameric transition, the Ser^191^Ala and Ala^191^Ser mutations were incorporated into the full-length *e*EI and *t*EI, respectively. Then, the ability of *e*EI, *e*EI_S191A_, *t*EI, and *t*EI_A191S_, to transfer the phosphoryl group from PEP to HPr was investigated at 25°C by ^1^H-^15^N SOFAST-TROSY spectra, as recently described (Nguyen et al., 2018). As expected, the data indicate that at room temperature *e*EI catalyzes the phosphoryl transfer reaction ∼10 times faster than *t*EI (Figure 5D). Interestingly, the Ser^191^Ala and Ala^191^Ser mutations produce no detectable changes in the activity of *e*EI and *t*EI, respectively (Figure 5D), indicating that the conformational transition from the *g* ^*+*^ to *g* ^*−*^ rotameric state of His^189^ is not rate limiting for catalysis.

## Discussion

Protein conformational transitions are fundamental to signaling, enzyme catalysis, and assembly of cellular structures. Yet, our understanding of how the interconversion among different folded structures affects function continues to lag. One technical challenge limiting our ability to interrogate the dynamics/function relationship is the lack of universal and straightforward strategies to selectively perturb conformational equilibria in complex biomolecular systems. Here, we have shown that it is possible to perturb protein conformational dynamics without dramatically affecting their thermal stability by hybridizing the amino acid sequence of a mesophilic and a thermophilic analogue. In particular, we have investigated the structure and dynamics of the N-terminal domain of EI from a mesophilic (*e*EIN) and a thermophilic (*t*EIN) bacterium. We found that the two proteins adopt the same fold and undergo a rotameric equilibrium at the His^189^ side chain that exposes the phosphorylation site to react with the EI substrate, PEP (Figure 1C). Interestingly, CPMG RD experiments revealed that the rotameric transition in *t*EIN occurs on a slower time scale than in *e*EIN (Figure 4A). By comparing the primary structures of the mesophilic and thermophilic proteins (Figures 5A,B) we identified a single point mutation in *e*EIN (Ser^191^Ala) and *t*EIN (Ala^191^Ser) that swaps the observed kinetics for this conformational change, with the *e*EIN_S191A_ mutant exchanging between the rotameric states of His^189^at a rate similar to the one measured for the wild type *t*EIC, and the *t*EIN_A191S_ mutant undergoing the same rotameric equilibrium on a faster time scale, comparable to wild type *e*EIN (Figure 4, Table 1). Intriguingly, we have recently used the same mesophilic/thermophilic hybridization strategy described here to engineer constructs of the C-terminal domain of EI (EIC) with modulated active site dynamics (Dotas et al., 2020). In contrast to the EIN case that allows for a single-point hybridizing mutation, design of the EIC hybrids required swapping of the entire active site (composed of three catalytic loops) between mesophilic and thermophilic species. Nonetheless, as for the EIN case presented here, the engineering effort resulted in production of two enzymatically active hybrids with mixed properties: One hybrid displayed the high thermal stability of the thermophilic enzyme and the increased active site flexibility and low-temperature activity of the mesophilic analogue; The second hybrid showed the low thermal stability of the mesophilic enzyme and the rigid active site and low activity at room temperature of the thermophilic protein (Dotas et al., 2020). Therefore, hybridizing homologue proteins from mesophilic and thermophilic bacteria is emerging as a powerful tool in biophysics by providing a straightforward approach to produce functional proteins with modulated internal flexibility.

In addition of serving as a demonstration of the mesophilic/thermophilic hybridization strategy for protein design, the EIN constructs engineered here allowed us to investigate the relationship between enzymatic turnover and the kinetics of the His^189^ rotameric equilibrium. Indeed, by introducing the hybridizing mutations at position 191 into the sequence of the full-length enzyme we have demonstrated that increasing the rate of the *g* ^*+*^-to-*g* ^*−*^ transition of the His^189^ χ2 angle does not affect turnover for the phosphoryl transfer reaction catalyzed by *t*EI (Figure 4, Figure 5). Similarly, increasing the activation energy for the His^189^ rotameric transition in *e*EI by introducing an Ala residue at position 191 does not affect its enzymatic activity (Figure 4, Figure 5). These results indicate that the His^189^ conformational change is not rate limiting for catalysis and, therefore, regulation of the EI activity cannot be achieved by slight perturbations of the His^189^ conformational dynamics.

Finally, it is important to highlight that the data reported in this manuscript show no evidence for an active state A/state B equilibrium in the isolated EIN. This observation implies that the latter equilibrium is either on a timescale that is not compatible with RD experiments (i.e., outside the μs-ms regime) or completely inactive in the isolated EIN domain. Considering that in the full-length dimeric EI transition to state B is required to avoid steric overlap between the EIN and EIC domains, and that state B is structurally stabilized by intersubunit EIN-EIN interactions (Figure 1A), we deduce that state B is inaccessible by the isolated EIN domain investigated here. In any case, our data indicate that the His^189^ *g* ^*+*^-to-*g* ^*−*^ transition that exposes the EI phosphorylation site to PEP (Figure 1C) is decoupled from the EIN state A/state B equilibrium and is not triggered by transition of the full-length enzyme to the catalytically active closed conformation (Figure 1A).

## Data Availability

The names of the repository/repositories and accession number(s) can be found below: BioMagResBank. Accession number: 50386
