# Supplementary material for: A Single Point Mutation Controls the Rate of Interconversion Between the g + and g − Rotamers of the Histidine 189 χ2 Angle That Activates Bacterial Enzyme I for Catalysis
Source: Front Mol Biosci. 2021 Jul 8;8:699203. doi: 10.3389/fmolb.2021.699203 (PMC8295985; doi:10.3389/fmolb.2021.699203)
Supplement: Supplementary file 1 [file DataSheet1.PDF]

Supplementary Information

A single point mutation controls the rate of interconversion between the g^+ and g^- rotamers of the Histidine 189 χ2 angle that activates bacterial Enzyme I for catalysis

Jeffrey A. Purslow[1], Jolene Thimmesch[1], Valeria Sivo[2], Trang T. Nguyen[1], Balabhadra Khatiwada[1], Rochelle R. Dotas[1] and Vincenzo Venditti[1,3,*]

[1] *Department of Chemistry, Iowa State University, Ames, Iowa 50011, USA.*

[2] *Department of Environmental, Biological and Pharmaceutical Sciences and Technologies, Università degli Studi della Campania, 81100 Caserta, Italy*

[3] *Roy J. Carver Department of Biochemistry, Biophysics and Molecular Biology, Iowa State University, Ames, Iowa 50011, USA.*

*Address correspondence to: Vincenzo Venditti, Department of Chemistry, Iowa State University, Hach Hall, 2438 Pammel Drive, Ames, IA 50011, USA. Email: venditti@iastate.edu; Tel: 515-294-1044; Fax: 515-294-7550; ORCID 0000-0001-8734-0400.

Supplementary Figures

X: active site X: conserved residues — EIN — EIC --- α-helix 6 *: mutation

```
eEI    1    MISGILASPGIAFGKALLLKEDEIVIDRKKISADQVDQEVERFLSGRAKASAQLETIKTK   60
tEI    1    MLKGVAASPGIAIGKAFLYTKEKVTINVEKIEESKVEEEIAKFRKALEVTQEEIEKIKEK   60

eEI    61   AGETFGEEKEAIFEGHIMLLEDEELEQEIIALIKDKHMTADAAAHEVIEGQASALEELDD   120
tEI    61   ALKEFGKEKAEIFEAHLMLASDPELIEGVENMIKTELVTADNAVNKVIEQNASVMESLND   120

eEI    121  EYLKERAADVRDIGKRLLRNILGLKIIDLSAIQDEVILVAADLTPSETAQLNLKKVLGFI   180
tEI    121  EYLKERAVDLRDVGNRIIENLLGVKSVNLSDLEEEVVVIARDLTPSDTATMKKEMVLGFA   180

                       *
eEI    181  TDAGGRTSHTSIMARSLELPAIVGTGSVTSQVKNDDYLILDAVNNQVYVNPTNEVIDKMR   240
tEI    181  TDVGGRTSHTAIMARSLEIPAVVGLGNVTSQVKAGDLVIVDGLEGIVIVNPDEKTVEDYK   240
                    --------------
                      α-helix 6
eEI    241  AVQEQVASEKAELAKLKDLPAITLDGHQVEVCANIGTVRDVEGAERNGAEGVGLYRTEFL   300
tEI    241  SKKESYEKKVEGLKQLKDLPAETPDGKKVMLAANIGTPKDVASALANGAEGVGLFRTEFL   300

eEI    301  FMDRDALPTEEEQFAAYKAVAEACGSQAVIVRTMDIGGDKELPYMNFPKEENPFLGWRAI   360
tEI    301  YMDRNSLPSEEEQFEAYKEVVEKMGGRPVTIRTLDIGGDKELPYLDMPKEMNPFLGYRAI   360

eEI    361  RIAMDRKEILRDQLRAILRASAFGKLRIMFPMIISVEEVRALRKEIEIYKQELRDEGKAF   420
tEI    361  RLCLDRPDIFKTQLRAILRASAYGNVQIMYPMISSVEEVRKANSILEEVKAELDREGVKY   420

eEI    421  DESIEIGVMVETPAAATIARHLAKEVDFFSIGTNDLTQYTLAVDRGNDMISHLYQPMSPS   480
tEI    421  DKEIKVGIMVEIPSAAVTADILAKEVDFFSIGTNDLTQYTLAVDRMNEHVKEYYQPFHPA   480

eEI    481  VLNLIKQVIDASHAEGKWTGMCGELAGDERATLLLLGMGLDEFSMSAISIPRIKKIIRNT   540
tEI    481  ILRLVKMVIDAAHKEGKFAAMCGEMAGDPLAAVILLGLGLDEFSMSATSIPEIKNIIRNV   540

eEI    541  NFEDAKVLAEQALAQPTTDELMTLVNKFIEEKTIC   575
tEI    541  EYEKAKEIAEKALNMSEAREIEKMMKDVI--KDIG   573
```

Figure S1. Sequence alignment of *e*EI and *t*EI. The amino acid sequences of *e*EI and *t*EI were aligned in BLAST. Active site residues are colored red. Conserved resides are colored green. The EIN and EIC domains are indicated with a blue and a red line underneath the sequence, respectively. The location of α-helix 6 is shown with a dashed black line underneath the amino acidic sequence. The single-point hybridizing mutation is indicated with an asterisk.

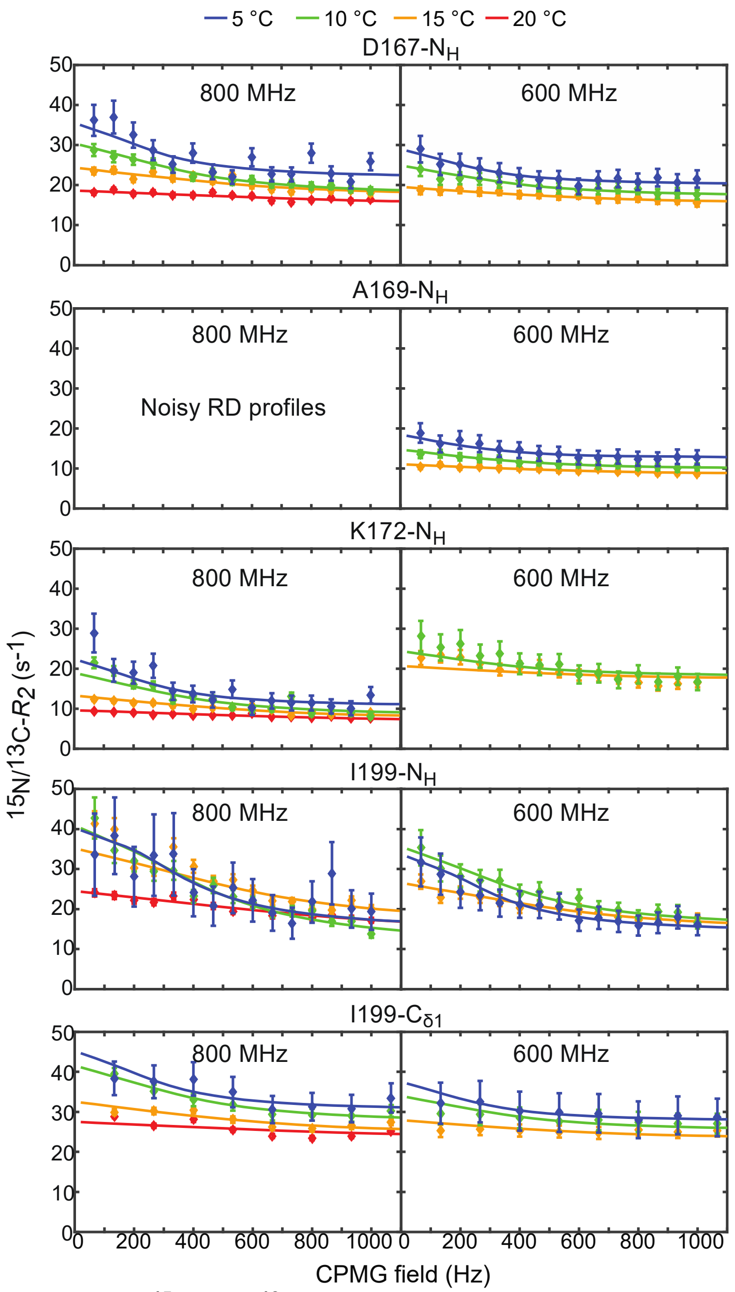

Figure S2. ^{15}N and ^{13}C$_{methyl}$ Relaxation Dispersion curves for *t*EIN. Global fitting of the ^{15}N and ^{13}C$_{methyl}$ relaxation dispersion curves measured at 20 (red), 15 (orange), 10 (green), and 5 (blue) °C and 800 (left panel) and 600 (right panel) MHz for *t*EIN. Experimental data are shown as circles. The global fit is shown as solid lines.

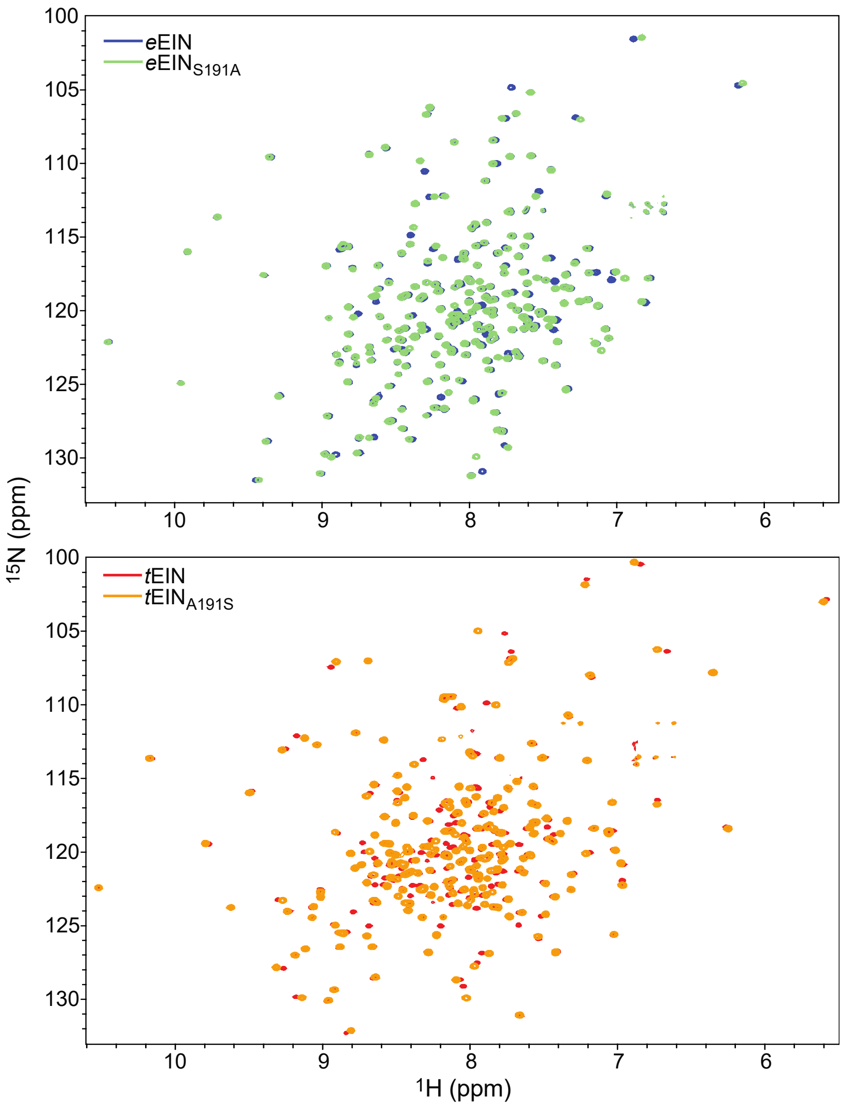

Figure S3. Effect of single point mutations on the structure of EIN. (**A**) 800 MHz ^{1}H-^{15}N TROSY of ^{15}N-labeled *e*EIN (blue) and *e*EIN$_{S191A}$ (green). (**B**) 800 MHz ^{1}H-^{15}N TROSY of ^{15}N-labeled *t*EIN (red) and *t*EIN$_{A191S}$ (orange).

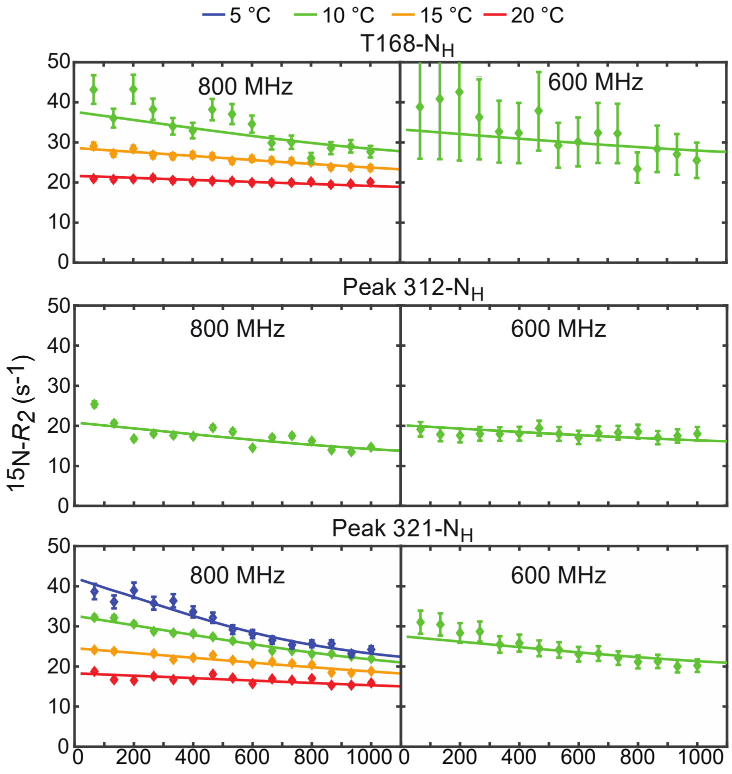

Figure S4. ^{15}N and ^{13}C$_{methyl}$ Relaxation Dispersion curves for *e*EIN$_{S191A}$. Global fitting of the ^{15}N and ^{13}C$_{methyl}$ relaxation dispersion curves measured at 20 (red), 15 (orange), 10 (green), and 5 (blue) °C and 800 (left panel) and 600 (right panel) MHz for *e*EIN$_{S191A}$. Experimental data are shown as circles. The global fit is shown as solid lines.
